# Identification of Novel *GRM1* Mutations and Single Nucleotide Polymorphisms in Prostate Cancer Cell Lines and Tissues

**DOI:** 10.1371/journal.pone.0103204

**Published:** 2014-07-25

**Authors:** Shafat Ali, Mojgan Shourideh, Shahriar Koochekpour

**Affiliations:** 1 Departments of Cancer Genetics, Center for Genetics and Pharmacology, Roswell Park Cancer Institute, Buffalo, New York, United States of America; 2 Departments of Urology, Center for Genetics and Pharmacology, Roswell Park Cancer Institute, Buffalo, New York, United States of America; Hormel Institute, University of Minnesota, United States of America

## Abstract

Metabotropic glutamate receptor 1 (GRM1) signaling has been implicated in benign and malignant disorders including prostate cancer (PCa). To further explore the role of genetic alterations of *GRM1* in PCa, we screened the entire human *GRM1* gene including coding sequence, exon-intron junctions, and flanking untranslated regions (UTRs) for the presence of mutations and single nucleotide polymorphisms (SNPs) in several PCa cell lines and matched tumor-normal tissues from Caucasian Americans (CAs) and African Americans (AAs). We used bidirectional sequencing, allele-specific PCR, and bioinformatics to identify the genetic changes in *GRM1* and to predict their functional role. A novel missense mutation identified at C1744T (582 Pro > Ser) position of *GRM1* gene in a primary AA-PCa cell line (E006AA) was predicted to affect the protein stability and functions. Another novel mutation identified at exon-intron junction of exon-8 in C4-2B cell line resulted in alteration of the *GRM1* splicing donor site. In addition, we found missense SNP at T2977C (993 Ser > Pro) position and multiple non-coding mutations and SNPs in 3′-UTR of *GRM1* gene in PCa cell lines and tissues. These novel mutations may contribute to the disease by alterations in *GRM1* gene splicing, receptor activation, and post-receptor downstream signaling.

## Introduction

Glutamate signaling leads to activation of multiple downstream signaling cascades. These downstream signaling cascades include (i) activation of ligand-gated ion channels (known as ionotropic glutamate receptors (iGluRs)) and influx of Ca^2+^ and/or K^+^ ions in central and peripheral nervous systems [1], (ii) activation of metabotropic glutamate receptors (mGluRs) known as G-protein coupled receptors (GPCRs) and second messenger pathways such as phospholipase C (PLC), phosphoinositide 3 kinase/retrovirus AK thymoma/mTOR (PI3K/AKT/mTOR) [2], and (iii) activation of G-protein-independent signal transduction pathways, mitogen activated protein kinase (MAPK) and protein kinase C (PKC)/ERK1/2 pathways [3], [4]. Last two signaling cascades, PI3K/AKT/mTOR and MAPK/ERK have extensively been implicated in multiple human cancers and emphasize the role of glutamate signaling in tumorigenesis [4].

mGluRs comprise a basic characteristic architecture: a large bi-lobed extracellular amino-terminal domain (ATD) also known as “venus fly trap” with a specific sequence of 24 amino acid for glutamate binding site, a 70 amino acid cysteine-rich domain (CRD) required for dimerization after activation, followed by classic seven alpha-helical transmembrane domain, and an intracellular cytoplasmic tail domain (CTD) [5]. Various isoforms of Group I mGluRs (mGluR1) have been identified depending on the length of C-terminal domain [5]. This C-terminal domain comprises a proline-rich Homer1 binding motif (isoform a), which involves in intricate protein-protein interactions and complex formation with downstream molecules [6]. Truncation of CTD leads to the loss of Homer1 binding motif in isoforms b-d and thus affects the interaction with other downstream signaling molecules and pathways [5]. Presence of different length isoforms of *GRM1* gene with varying role in subsequent activation of downstream signaling pathways, highlight the importance of splicing mechanisms in *GRM1* gene function [5].

mGluRs signaling was initially implicated in cellular proliferation of glioma cells [7] and melanoma development [8] either in *in vitro* or *in vivo* studies and subsequently this receptor was shown to play a crucial role in various types of cancers [9], [10], [11], [12] Oncogenic function of mGluR1 was shown by the induction of transformed phenotype with overexpression of *GRM1* gene in melanocytes [13]. Similarly, in our previous study, we investigated the expression of mGluR1 in primary and metastatic PCa cell lines and tissues [10]. PCa cells growth dependence on *GRM1*-signalling was also demonstrated by glutamate blockade or use of Riluzole, as a GRM1 antagonist or an inhibitor of glutamate release. Riluzole reduced the PCa cells proliferation, migration, invasion, and induced apoptosis as shown by increased expression level of cleaved caspase-9, -7, -3, PARP, and phopho-H2AX^ser139^ [10].

Somatic mutations have been identified in different domains of mGluRs, including ligand-binding domain, transmembrane domain and cytosolic domain in various types of cancer [4]. Missense mutations of mGluR identified in different domains might have biological relevance in cancers. In fact, somatic mutations in the mGluRs have been reported to promote breast cancer, melanoma, and renal cell carcinoma growth and progression *in vivo* [11], [12], [14]. Esseltine and colleague [15] studied the functional consequences of multiple missense mutations identified in different domains of mGluRs in several types of cancers including lung adenocarcinoma, squamous cell carcinoma, high-grade astrocytoma, and breast cancers. These missense mutations in different domains of mGluRs affect receptor expression and provide selective advantage to different downstream signaling pathways (e.g., PI3k/Akt/mTOR, ERK1/2 activation) and changes in cellular morphology.

Few reports exist for GRM1 mutations and single nucleotide polymorphisms (SNPs) in PCa cell lines or tissues. These studies are limited to whole-exome sequencing and transcriptome analysis. These high throughput assays are unable to identify intron-exon junctions or non-coding variants and require further experimental validation of the identified mutations. In addition, these studies did not include African American (AA)-PCa samples and cell lines for GRM1 mutation or SNP detection. To overcome these limitations, we screened full-length GRM1 gene including all exons, exon-intron junctions, and flanking non-coding 5′- and 3′-untranslated regions (UTRs) to identify genetic alterations in several PCa cell lines and matched tumor-normal tissues in AAs and Caucasian Americans (CAs). We identified novel mutations and SNPs in GRM1 gene. Functional consequences of these mutations were predicted using available online bioinformatics tools.

## Materials and Methods

### Cell Lines

We screened the genomic DNA of ten PCa cell lines (PC-3, E006AA, DU-145, MDA-PCa2b, 22RV1, LAPC4, VCaP, CWR-R1, LNCaP, C4-2B) and normal prostate epithelial cell for detection of mutations and polymorphisms in coding region, flanking untranslated regions (5′- and 3′- UTRs), and exon-intron junctions of full length *GRM1* gene.

Castrate-resistant (PC-3, DU-145, MDA-PCa2b, VCap, and 22RV1) and androgen- stimulated (LNCaP and LAPC4) PCa cell lines were purchased from American Type Culture Collection (ATCC, Manassa, VA) [16], [17]. Normal prostate epithelial cells (NL-Pr.EC) and C4-2B cell lines were purchased from Clonetics (Bio Whittaker, Walkersville, MD) and UROCOR (Uroscience Group, Oklahoma City, OK) respectively. E006AA was established from an AA patient with organ-confined PCa [16]. CWR-R1 cell line was provided as a gift from Dr. Elizabeth Wilson (University of North Carolina, Chapel Hill, NC, USA) [18]. PC-3, DU-145, and VCaP cell lines were maintained in DMEM supplemented with Fetal Bovine Serum (FBS, 10%) and antibiotics (1%). E006AA, 22RV1, CWR-R1, C4-2B, and LNCaP cell lines were cultured in RPMI1640 with FBS (10%) and antibiotic (1%). MDA-PCa2b cell line was cultured in define medium as recommended by the manufacturer (ATCC, Manassa, VA). LAPC4 cell lines was culture in IMDM supplemented with 10% FBS and 1% antibiotic.

### Tumor Samples and Ethical Statement

To evaluate genetic alterations in *GRM1* gene, we included a total of 21 matched prostate normal-tumor samples obtained from AAs (n = 10) and CAs (n = 11). All tissues biospecimens were obtained from biospecimen core facility at the Louisiana Cancer Research Consortium (LCRC) affiliated to Tulane Medical School and School of Medicine, Louisiana State University Health Sciences Center (LSUHSC, New Orleans, LA). Pre-informed written consent form was obtained from each patient during samples collection for use of their samples in scientific discoveries and the study was approved by Institutional Review Board (IRB) at LSUHSC. Matched normal-tumor tissues were available for eight cases (N3-T3; N4-T4; N5-T5; N13-T13; N21-T21; N35-T35; N52-T52; N71-T71) of CAs and six cases (N2-T2; N6-T6; N26-T26; N27-T27; N32-T32; N38-T38) of AA patients.

### Genomic DNA Isolation, Polymerase Chain Reaction, and Sequencing of *GRM1* Gene

Genomic DNAs were isolated from prostate tissues and cell lines using AllPrep DNA/RNA Quigen kit (Qiagen, Valencia, CA). Full-length *GRM1* gene (isoform α) was selected (NM_000838.3) to design the primers covering all exons, exon-intron junctions, and 5′- and 3′-UTRs. This transcript comprises total of nine exons. Based on mutations and SNPs discovered in exon-8 and -9 in PCa cell lines, we selected these regions for further sequencing in matched tumor-normal tissues. Primer designing was performed using PrimerSelect tool of DNASTAR lasergene 9 core suit (DNASTAR, Madison, WI). A total of 9492 bp was amplified in sixteen amplicons including the flanking 50–100 bp covering the exon-intronic sequence. Primer details (sequence, melting temperature, and amplicon size) are provided in Table S1. Each genomic DNA sample (total 25 ng) was amplified by 32 cycles in 10 µl total volume of polymerase chain reaction (PCR) containing specific primers (0.2 µM), dNTPs (0.2 mM), MgCl2 (1.5 mM); GoTaq DNA polymerase (0.75 U) in 1x PCR Buffer (Promega, Madison, WI, USA). PCR products were purified by treating with 5 U of Exonuclease-I (Exo-1) and 2 U of Shrimp Alkaline Phosphatase (SAP) along with 1x buffer at 37 oC for 2–3 hours followed by heat-inactivation of enzymes at 85 oC for 20 minutes. After Exo-SAP treatment, PCR products were diluted to 15 ng/µl concentration. PCR product specificity, quantity, and quality were analyzed by 1.2% agarose gel electrophoresis. Bi-directional sequencing of the PCR products was performed at Genomic core facility (RPCI, Buffalo) using ABI 3130xl Genetic Analyzer (Applied Biosystems, CA). Data were analyzed using sequence analysis software and verified by visual examination of the identified variants in electropherogram.

### Allele-Specific Genotyping of C1744T Mutation in Tumor Samples

We developed an allele-specific PCR-based genotyping method to evaluate the non-synonymous mutation identified by direct sequencing of *GRM1* gene at C1744T (582 Pro>Ser) position in E006AA cell line. For bi-directional amplification of this specific mutation, four primers were designed (Table S1, Primer numbers 19 to 22), two outer primers and two allele-specific (AS) inner primers with their 3′- end specificity to each allele (C>T). C-allele is amplified in one direction with an AS inner primer and an outer primer pair (#19 and #20; amplicon size 439 bp), whereas mutant T-allele is amplified in the opposite direction with the other inner and outer primer pair (#21 and #22; amplicon size 297 bp). The two outer primers also amplify a constant fragment (#20 and #22; amplicon size 736 bp), which serve as positive controls for PCR. All four primers were used in single reaction and PCR conditions were optimized by adjusting the concentration of each primer and annealing temperatures (Table S1). In every set of PCR reaction, we included a positive and a negative control sample to observe the efficiency of the AS-genotyping.

### Bioinformatic Analysis of Identifies Novel *GRM1* Mutations

Novel mutations identified in *GRM1* gene were tested bioinformatically for different possible effects on protein structure, function, and splice site variant. The effect of missense mutation (serine to proline) at 582 aa position was tested using online tools; (i) PolyPhen2 (http://genetics.bwh.harvard.edu/pph2/) [19], (ii) SIFT (http://sift.jcvi.org/) [20], and (iii) Phd-SNP (http://gpcr2.biocomp.unibo.it/cgi/predictors/PhD-SNP/PhD-SNP.cgi) [21].

To predict the potential structural consequence of the identified mutation at exon-intron junctions of exon-8 in *GRM1* gene, we used two different online bioinformatics tools: (a) Human splicing finder (http://www.umd.be/HSF/) [22] and (b) ESEfinder (http://rulai.cshl.edu/tools/ESE/) [23]. DNA sequence comprising wild type or mutant allele was used as a query in Human Splice Finder tool to predict the splicing donor or acceptor site.

## Results

### Identification of Novel *GRM1* Mutations and SNPs in prostate cancer cell lines

**Figure 1:**
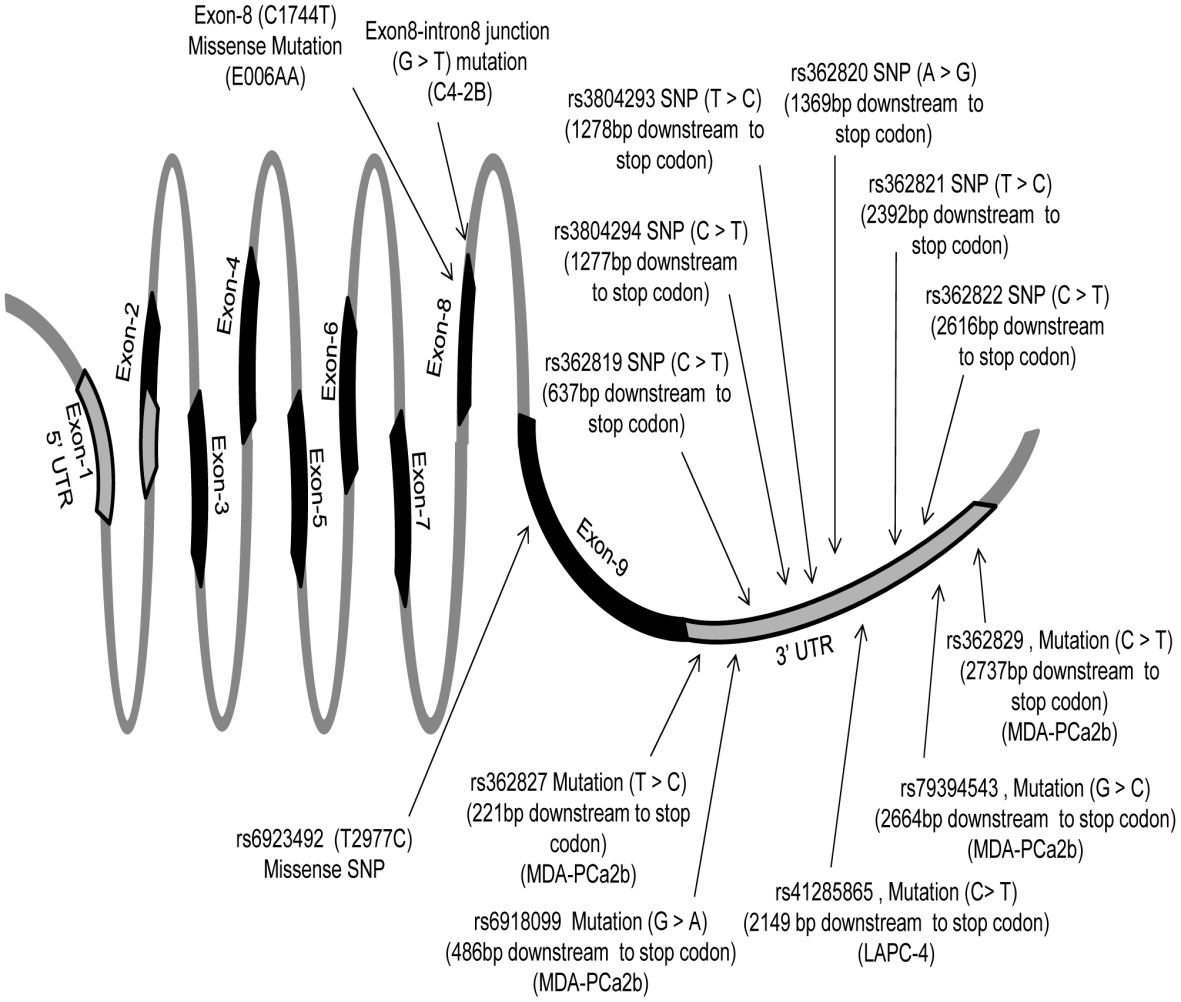
Schematic presentation of the identified mutations and polymorphisms of full-length *GRM1* gene in prostate cancer cell lines and tissues.

**Figure 2:**
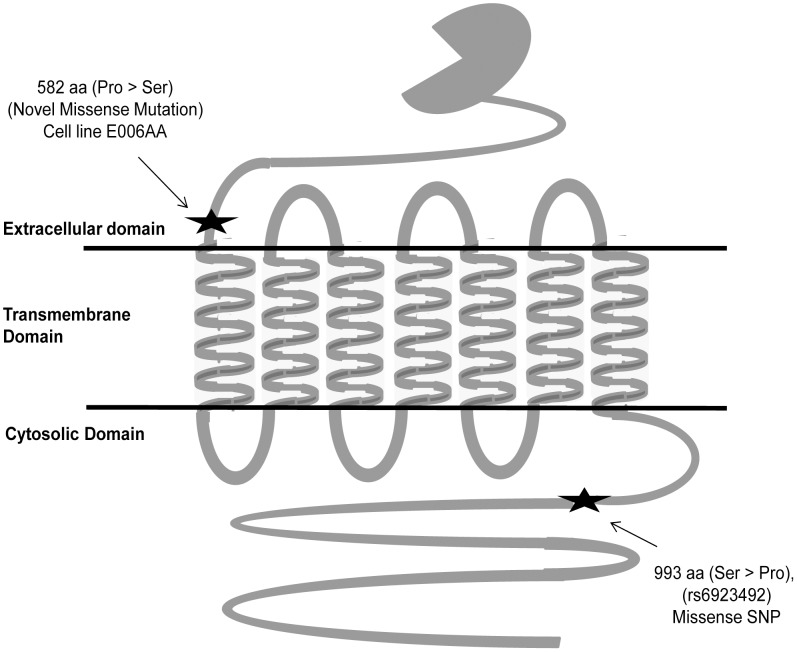
Location of the novel identified mutations and polymorphisms in different domains of GRM1 receptor.

Sequencing of whole exons of *GRM1* gene in 10 PCa cell lines showed 18 genetic alterations that include newly identified non-synonymous mutations, splice-site variations, non-synonymous, synonymous, and non-coding polymorphisms (Figure 1 and 2; Table 1–2). A novel non-synonymous mutation at C1744T (582 Pro>Ser) position of exon-8 was detected in E006AA, a primary AA-PCa cell line (Figure 1). This mutation was appeared in heterozygous status (CC>CT) and located close to transmembrane domain at extra cellular side of GRM1 receptor and resulted in proline (hydrophilic amino acid, codon-CCT) to serine (neutral amino acid, codon-TCT) amino acid change (Figure 2). Second novel mutation was identified at exon-intron junction of exon-8 in C4-2B, a castrate-resistant PCa cell line (Figure 1, Table 1). This mutation resulted in G to T conversion at the start of intron-8 and appeared in heterozygous condition. Five non-coding mutations were also found in 3′-UTR region of exon-9 of *GRM1* gene in two different PCa cell lines (LAPC4 and MDA-PCa2b) (Table 2). One of these mutations is located at 2149 bp downstream of stop codon in LAPC4 and reported in NCBI data base (dbSNP build 139) as rs41285865. While other four mutations located at 221 bp, 486 bp, 2664 bp and 2737 bp downstream to stop codon in MDA-PCa2b cell line (Table 2). These mutations reported in NCBI data base (dbSNP build 139) as rs362827, rs6918099, rs79394543 and rs362829 respectively.

**Table 1:** Genotype status of non-synonymous and synonymous mutations and polymorphisms identified in *GRM1* gene in prostate cancer cell lines.

	Exon-8		Exon-9				
			Missens*e* SNP	Synonymous SNPs			
Cell lines	(582 aa) a (C>T), Mis b (Pro>Ser)	Exon –Intron junctions (G>T) mutation	rs6923492; 993aa (T>C) (Ser > Pro)	rs2942; 931aa (G>A) (Lys)	rs6923864; 1056aa (T>G), (Gly)	rs1047006; 1071aa (T>G), (Pro)	rs9373491; 1165aa (C>A), (Pro)
**PC-3**	CC	GG	TT	GG	TT	TT	CC
**E006AA**	**CT**	GG	CC	AA	GG	GG	AA
**DU-145**	CC	GG	CC	AA	GG	GG	AA
**MDA-PCa2b**	CC	GG	TC	GG	GG	TT	CA
**22RV1**	CC	GG	TT	GG	TT	TT	CC
**LAPC4**	CC	GG	CC	AA	GG	TG	AA
**VCap**	CC	GG	TC	AG	TG	TT	CC
**CWR-R1**	CC	GG	TT	GG	TT	TT	CC
**C4-2B**	CC	**GT**	CC	AA	GG	GG	AA
**LNCaP**	CC	GG	CC	AA	GG	GG	AA
**NL-Pr.EC**	CC	GG	TT	GG	TT	TT	CC

a Novel mutations identified in our study.

b Mis: Missense mutation.

**Table 2:** Genotype status of downstream non-coding polymorphisms identified in *GRM1* gene in prostate cancer cell lines.

	Exon-9 SNPs (DSSC) a										
Cell lines	rs362827 (221bp)	rs6918099 (486bp)	rs362819 (637bp)	rs3804294 (1277bp)	rs3804293 (1278bp)	rs362820 (1369bp)	rs41285865 (2149)	rs362821 (2392bp)	rs362822 (2616bp)	rs79394543 (2664bp)	rs362829 (2737bp)
**PC-3**	TT	GG	TT	TT	GG	GG	CC	TT	CC	GG	CC
**E006AA**	TT	GG	CC	CC	TT	AA	CC	CC	TT	GG	CC
**DU-145**	TT	GG	CC	CC	TT	AA	CC	CC	TT	GG	CC
**MDA-PCa2b**	**TC**	**GA**	CC	CC	GT	AG	CC	TT	CC	**GC**	**CT**
**22RV1**	TT	GG	TT	TT	GG	GG	CC	TT	CC	GG	CC
**LAPC4**	TT	GG	CC	CC	TT	AA	**CT**	CC	TT	GG	CC
**VCap**	TT	GG	TT	CC	TT	AG	CC	CT	CT	GG	CC
**CWR-R1**	TT	GG	TT	TT	GG	GG	CC	TT	CC	GG	CC
**C4-2B**	TT	GG	CC	CC	TT	AA	CC	CC	TT	GG	CC
**LNCaP**	TT	GG	CC	CC	TT	AA	CC	CC	TT	GG	CC
**NL-Pr.EC**	TT	GG	TT	TT	GG	GG	CC	TT	CC	GG	CC

a DSSC: Downstream to stop codon.

Apart from these mutations, one missense SNP (rs6923492) was identified at 993 amino acid position in cell lines investigated. Homozygous TT genotype was observed in PC-3, 22RV1, CWR-R1 cell lines, and NL-Pr.EC. Heterozygous TC genotype was discovered in MDA-PCa2b and VCaP cell lines, and homozygous CC genotype in E006AA, DU-145, LAPC4, C4-2B, LNCaP cell lines. At this locus, change of T to C nucleotide resulted in Serine (TCC) to Proline (CCC) conversion. This missense polymorphism is located in cytoplasmic domain of GRM1 receptor between transmembrane domain and coiled-coil motif (Figure 2). Four previously reported synonymous polymorphisms were also identified in coding sequence of exon-9 at 931 (rs2942), 1056 (rs6923864), 1071 (rs1047006) and 1165 amino acid (rs9373491) positions (Table 1). Six non-coding SNPs were found in 3′-UTR of *GRM1* gene and these polymorphisms are located at 637 bp (rs362819), 1277 bp (rs3804294), 1278 bp (rs3804293), 1369 bp (rs362820), 2392 bp (rs362821) and 2616 bp (rs362822) downstream to stop codon (Table 2).

### Identification of *GRM1* Mutations and SNPs in Prostate Cancer Tissues

Sequencing of selected exons-8 and -9 of *GRM1* gene in prostate tumor tissues showed multiple genetic alterations. A homozygous genotype status (CC genotype) compared to heterozygous genotype (CT genotype) in adjacent normal tissue at 637 bp downstream (rs362819) to stop codon was found in an AA tumor (N38-T38 samples, Table 3). Another two genotypes (CC and GA) were found in 3′-UTR region only in AA samples at position 221 bp (rs362827; N2-T2) and 486 bp (rs6918099; N6-T6, N32-T32 and N38-T38) downstream to stop codon. However, these changes were of germline origin. Since the heterozygous genotype (GA at rs6918099) at 486 bp downstream to stop codon was found in 3 out of 11 AAs, it appears to be an ethnic-specific genotype. The homozygous CC genotype (rs362827) that exists only in a single AA-patient may be a sporadic germ line mutation or a rare variant.

**Table 3:** Genotype status of mutations and SNPs identified in *GRM1* gene in matched prostate tumor-normal tissues.

			Exon-9 Variations											
			Missense SNP		Synonymous SNPs				DSSC a					
Matched normal-tumor samples			rs6923492 (993aa) TT (Ser); CC (Pro)		rs2942, 931aa(G>A) Lys > Lys		rs6923864, 1056aa(T>G), Gly>Gly		rs362827 (T>C) (221bp)		rs6918099 (486bp)		rs362819 (637bp)	
Ancestry	Normal	Tumor	Normal	Tumor	Normal	Tumor	Normal	Tumor	Normal	Tumor	Normal	Tumor	Normal	Tumor
**African Americans**	N-2	T-2	CC	CC	GG	GG	GG	GG	CC	CC	GG	GG	CC	CC
	N-6	T-6	CT	CT	AG	AG	GG	GG	TT	TT	GA	GA	CC	CC
	N-26	T-26	CC	CC	AG	AG	GG	GG	TT	TT	GG	GG	CC	CC
	N-27	T-27	CC	CC	AA	AA	GG	GG	TT	TT	GG	GG	CC	CC
	N-32	T-32	CC	CC	AG	AG	GG	GG	TT	TT	GA	GA	CC	CC
	N-38	T-38	CT	CT	GG	GG	TG	TG	TT	TT	GA	GA	CT	CC
	N/A	T-62		CC		AA		GG		TT		GG		CC
	N/A	T-25		CC		AA		GG		TT		GG		CC
	N/A	T-85		CT		AG		GG				GG		CC
	N/A	T-11		CC		AA		GG				GG		CC
**Caucasian Americans**	N-3	T-3	CC	CC	AA	AA	GG	GG	TT	TT	GG	GG	CC	CC
	N-4	T-4	TT	TT	GG	GG	TT	TT	TT	TT	GG	GG	TT	TT
	N-5	T-5	CC	CC	AA	AA	GG	GG	TT	TT	GG	GG	CC	CC
	N13	T13	CT	CT	AG	AG	TG	TG	TT	TT	GG	GG	CC	CC
	N21	T21	CC	CC	AA	AA	GG	GG	TT	TT	GG	GG	CC	CC
	N35	T35	CT	CT	AG	AG	TG	TG	TT	TT	GG	GG	CC	CC
	N52	T52	CT	CT	AG	AG	TG	TG	TT	TT	GG	GG	CC	CC
	N71	T71	CT	CT	AG	AG	TG	TG	TT	TT	GG	GG	CC	CC
	N/A	T7		TT		GG		TT		TT		GG		TT
	N/A	T20		CC		AA		GG		TT		GG		CC
	N/A	T34		CC		AA		GG		TT		GG		CC

a DSSC: Downstream to stop codon.

Similar to cell line sequencing data, we identified a missense polymorphism (rs6923492) at 993 amino acid position leading serine to proline amino acid change and was observed in 7 out of 10 AAs (N2-T2, N26-T26, N27-T27, N32-T32, T62, T25, T11) and 5 out of 11 CAs (N3-T3, N5-T5, N21-T21, T20, T34) tissues (Table 3). A non-coding SNP (rs362819) was found at 637 bp downstream to stop codon. The genotype status for these SNPs in tumor tissues was same to adjacent normal tissue, indicating germ line polymorphisms (Table 3). Two synonymous polymorphisms were also found in exon-9 in both AAs and CAs populations. These were located at 931 amino acid position (rs2942) and code for lysine residue, while another synonymous polymorphism located at 1056 amino acid position (rs6923864) code for glycine residue.

**Figure 3:**
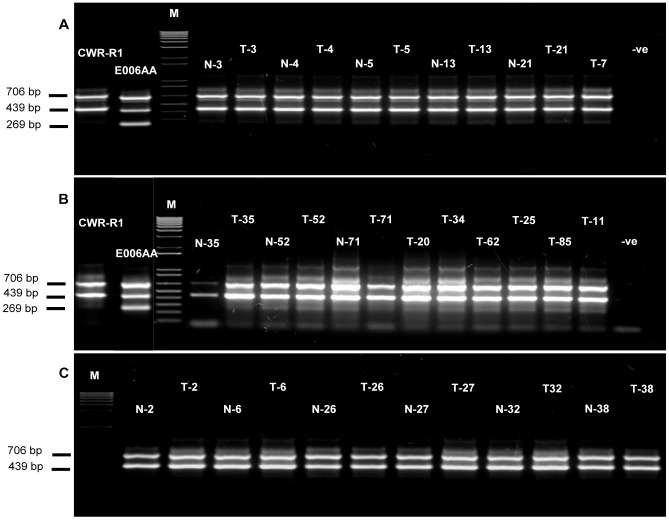
Allele-specific PCR genotyping of novel missense mutation identified in exon-8 of *GRM1* gene in prostate tumors. Presence of C-allele showed amplification of a 439 bp fragment and presence of T-allele showed amplification of a 267 bp fragment. A nonspecific fragment of 706 bp was amplified in all samples.

Using allele-specific (AS) PCR primers and direct sequencing in matched tumor-normal tissues, we genotyped the newly identified non-synonymous mutation at C1744T (582 Pro>Ser) position (in E006AA) and the mutation at exon-intron junction of exon-8 (in C4-2B). These mutations were not detected in any of the malignant prostate tissues (**Figure 3**).

### Predicted Functions of the Identified *GRM1* mutations

The prediction of functional role of novel non-synonymous (582 Pro>Ser) mutation identified in *GRM1* was carried out using different tools. PolyPhen-2 predicted a deleterious Bayes posterior probability score value 0.60 for this mutation. This prediction score is based on multiple sequence alignment with homologous sequence of this gene in database and indicate that the mutation affects the protein stability or its function.

Independently analyzed, Sorting Intolerant From Tolerant (SIFT) algorithm also predicted an intolerance score value 0.02 for amino acid substitution identified at this locus. Based on protein sequence conservation throughout the evolution, SIFT predicted that 582 Pro>Ser amino acid substitution may also affect protein function. Furthermore, we used PhD-SNP method that utilizes available disease-related SNP database and machine learning technique to predict nsSNP related to human disease. This analysis revealed disease-relevant of the 582 Pro>Ser mutation with a reliability index value of 3.

**Figure 4:**
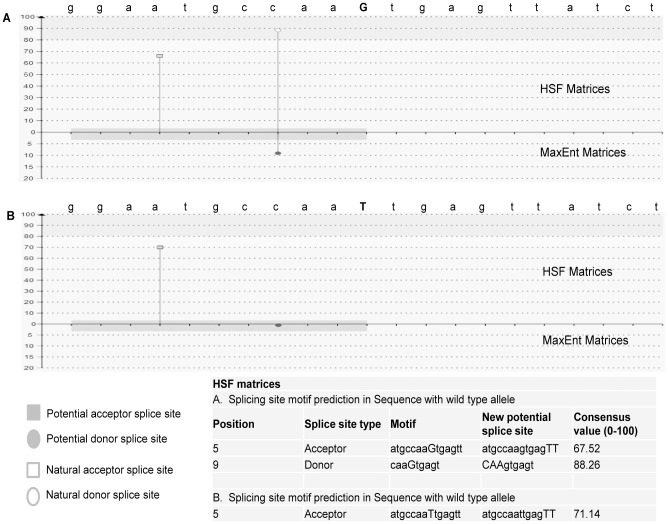
Bioinformatics prediction of splicing site motif (donor/acceptor). Twenty one base pair sequence flanking of mutation site with wild type (**A**) and mutant allele (**B**) was tested for prediction of splicing site motif (donor or acceptor).

Since point mutations at exon-intron or intron-exon junction may lead to exon slipping or alternative splicing, we were interested to analyze the potential effect of the novel mutation discovered at exon-intron junction of exon 8 in C4-2B cell line. For this, we employed the Human Splicing Finder (HSF) and Exon Splicing Enhancer (ESE) finder tools to predict the consequence on splice site motif with two different alleles of identified mutation [22], [23]. We found that the presence of T-allele strongly predicts the possibility of splice donor motif (caaGtgagt) with a high consensus value of 88.26. However, substitution of T- to G-allele resulted in loss of this splice donor motif (Figure 4A and 4B). Likewise, the ESEfinder predicted a high score (9.49) motif (exonic-splicing enhancer) for sequence with G-allele at the exon 8-intron junction of *GRM1* gene. However, this score reduced to -7.52 for motif sequence with mutant T-allele.

## Discussion

GRM1 signaling has been implicated in multiple malignant cancers including colon adenocarcinoma, melanoma, lung carcinoma, thyroid carcinoma, breast carcinoma, astrocytoma, neuroblastoma and rabdomyosarcoma [4], [5], [24]. Glutamate receptor gained a particular interest given its transforming potential and the availability of its ligand, glutamate, in the context of tumor microenvironment and easily accessibility of receptor at the surface of tumor cells. Differential expression, activation status or signal reprogramming of glutamate receptor in tumor cells can contribute to cancer development and progression. These alterations may arise from specific changes including somatic and germline genetic alterations, copy number variation, epigenetic, and post-translational changes resulting in altered expression or activation and thereby enhancing tumor progression and metastasis [25].

Deep sequencing studies have shown that G-protein coupled receptor (GPCR) are mutated in approximately 20% of all cancer and glutamate receptor family represent a second most frequently mutated GPCRs family member [26]. Somatic mutations in *GRM1* has been reported in wide varieties of tumors such as melanoma, breast carcinoma, colon carcinoma, lung adenocarcinoma, brain tumors, heamatopoitic, and lymphoid tissue [15]. *GRM1* gene comprises of different domains with specific functions, including ligand-binding domain, cysteine-rich domain for dimerization, transmembrane, and C-terminal domain for interaction with downstream signaling molecules. Mutations in these conserved residues might play a possible role in binding of GRM1 receptor with ligand, signaling initiation, transmission of signal, or termination or gain of function phenotypes. These mutations may not only involve in tumor development and progression but also affect metastasis including cell motility and promotion of angiogenesis.

Previous studies have shown that mutations in different G-protein coupled receptor, CCK2R and GRM1 are associated with altered receptor function, downstream signaling pathways, altered cellular morphology, migration, and promote angiogenesis, leading to enhanced tumerogenesis [15], [27]. Esseltine *et al* studied the functional role of *GRM1* mutations located at glutamate-bonding site (A168V), glutamate-binding domain (R375G), cysteine-rich region (G396V), G-protein binding region (R696W), and homer binding region (P1148L) in the carboxy-terminal tail of GRM1 receptor [15]. Transient expression of the mutated *GRM1* was associated with either reduced cell surface expression or basal/quisqualate-stimulated activation of inositol phosphate (IP) and/or ERK1/2 activation [15]. Mutation in homer-binding region (P1148L) was shown to alter cellular morphology and might affect cellular migration.

Although multiple mutations have been reported in *GRM1* gene in different human cancers [4], only a limited number of high-throughput studies including whole exome sequencing or transcriptome analysis were carried out and identified few *GRM1* mutations in PCa samples [28], [29]. Two novel mutations (R868H and G144S) in *GRM1* (Table S2) were identified by sequencing the exomes of 50 lethal catrate-resistant PCa, 11 high-grade primary organ-confind PCa samples (treatment naïve) and 11 PCa cell lines [29]. Exome sequencing of 112 prostate tumor/normal pairs identified two missense mutations (A573E and R681H) in *GRM1* gene [28]. A nonsense mutation was also reported in exon-2 of *GRM1* gene (TCGA: https://tcga-data.nci.nih.gov/tcga/).

Exome or transcriptome sequence analysis have their inherent limitations including the inability to detect mutations in exon-intron junctions or noncoding region and the need for further experimental validation of the identified mutations [28]. Further, these studies did not include the AA-tumor samples or their cell lines. Due to these limitations, we screened, for the first time, the entire exonic region of *GRM1* gene including flanking 5′- and 3′-UTRs and exon-intron junctions in ten commonly used PCa cell lines including the two established AA-PCa cell lines (E006AA and MDA-PCa2B), and 21 matched prostate tumor-normal tissues from 11 CAs and 10 AAs patients.

In this study, we identified two novel mutations in E006AA and C4-2B cell lines. These mutations were not identified in previous studies based on their study design or the cell lines included for sequencing [29]. Non-synonymous mutation (C to T) at 582 amino acid position resulted in proline to serine change. This mutation is located near transmembrane domain at extracellular side. Bioinformatics prediction of functional role of this mutation, using multiple analytical tools, showed the conversion of hydrophilic (Serine) to neutral (Proline) amino acid is either intolerant or leads to deleterious effect to protein stability and function. A conversion from serine to proline amino acid may also affect the strength of signal transmission after ligand binding and GRM1 receptor activation. Another mutation identified in exon-intron boundary of *GRM1* exon-8 in C4-2B cell line.

Bioinformatics analysis using two different online tools predicted that G to T nucleotide conversion at this position resulted in disruption of splicing donor site. Disruption of splicing-site motif at exon-intron junction at exon-8 position may provide the basis for creating different splice variants in *GRM1* gene. Various isoforms of *GRM1* gene with different protein length are associated with differential activation of downstream signals either through changes in the signal strength or interaction with other cytosolic proteins [30]. Further studies are required to validate the functional role of novel mutations of *GRM1* gene identified in this study and those by other investigators [28], [29]. Four additional mutations were identified in MDA-PCa2b and one mutation in LAPC4 cell line located in 3′-UTR of *GRM1* gene and these mutations have been reported in NCBI data base (dbSNP Build 39) in different populations. These mutations occurred as germline alterations in tumor samples investigated and we observed that the frequency of these mutations is high in AA tumor samples as compared to CAs. High frequency of these mutations observed in AA samples may associate with differential *GRM1* expression and function, which may result in the progression or more severity of the disease. AA population has shown a high incidence and mortality rate and presents a clinically more aggressive disease than CAs. The functional role of these mutations has not been reported in literature [31].

A missense polymorphism (rs6923492 T>C) identified in exon-9 of *GRM1* gene in PCa cell lines and tumors resulted in serine to proline change at 993 amino acid position at cytoplasmic end of receptor. Previous study using 1000 breast cancer patients showed association of this SNP in ER+/PR+ ductal carcinoma in TT-genotype carrier with later age at diagnosis (4.9 years) as compared to either TC- and CC-genotype carriers [32]. However, no association was found with melanoma susceptibility [33]. A larger tumor samples study is required to confirm the association of this SNP with susceptibility to prostate carcinogenesis, aggressiveness and progression.

## Conclusions

In this study, we identified novel mutations in *GRM1* gene in addition to previously reported mutations and SNPs in PCa cell lines and also in a subset of malignant prostate tissues. These novel mutations were predicted to play an important role in gene function including protein stability and splicing of different isoforms. Functional validation of these mutations will further strengthen the role of genetic alterations of *GRM1* gene in prostate carcinogenesis and progression.

## Supporting Information

Table S1 **Details of primers used for sequencing of** ***GRM1*** **gene.** Genomic sequences of primers used for PCR and sequencing in accordance with Human Genome Assembly (*GRM1* gene accession # NM_000838.3).
(DOC)

Table S2 **Details of somatic mutations in** ***GRM1*** **gene identified in whole exome or transcriptome sequencing studies of prostate cancer cell lines and tumors.**
(DOC)
