# Supplementary material for: Identification of Novel GRM1 Mutations and Single Nucleotide Polymorphisms in Prostate Cancer Cell Lines and Tissues
Source: PLoS One. 2014 Jul 25;9(7):e103204. doi: 10.1371/journal.pone.0103204 (PMC4111546; doi:10.1371/journal.pone.0103204)
Supplement: Table S1 — Details of primers used for sequencing of GRM1 gene. Genomic sequences of primers used for PCR and sequencing in accordance with Human Genome Assembly (GRM1 gene accession # NM_000838.3). (DOC) [file pone.0103204.s001.doc]

**Table S1:** Details of primers used for sequencing of *GRM1* gene**^1^**.

| **S.N.** | **Location ^2^** | **Primer Sequence (5’ – 3’)** | **Tm** | **Amplicon Size (bp)** |
| --- | --- | --- | --- | --- |
| 1 | Exon 1 Forward (5’-UTR) | CCCCTCCTCCCTCTATCAAACGAC | 64 | 377 |
| 2 | Exon 1 Backward (5’-UTR) | CTGGGACCCGGAGGAAAATCTG |
| 3 | Exon 2-1 Forward | CCCCAGAGTTTTAACACAGGTC | 57 | 735 |
| 4 | Exon 2-1 Backward | CCAGAGCCACGGAAGAGT |
| 5 | Exon 2-2 Forward | AGCCCTCTTCTCAGTCCATCA | 57 | 663 |
| 6 | Exon 2-2 Backward | TCCCCACAGTGCTAGAAATAAACA |
| 7 | Exon 3 Forward | AAATTATTTGGCAAGTCCGTTCTCT | 60 | 455 |
| 8 | Exon 3 Backward | GCAAGCATATGTGTTTCAAAGTGTG |
| 9 | Exon 4 Forward | GCCTTTTCTGGATTGCTTCATTC | 60 | 426 |
| 10 | Exon 4 Backward | AGCCCTGGCACCTTCTATTCTCAT |
| 11 | Exon 5 Forward | CCTTCCTCTGAGAACCCCCAAAAG | 70 | 453 |
| 12 | Exon 5 Backward | TATCCTCATGGCACCCAGAAACCA |
| 13 | Exon 6 Forward | ATACACATTTGGGATAATCAGAGG | 60 | 459 |
| 14 | Exon 6 Backward | CCTGGCACACCCTTTCTAGACAG |
| 15 | Exon 7 Forward | CCAAACCCAGCATGGAATGA | 57 | 387 |
| 16 | Exon 7 Backward | AAGGCAAATAGTGAGGCACAAGGTT |
| 17 | Exon 8-1 Forward | TGCAAAGATGACTGTGTCTCTGGT | 65 | 724 |
| 18 | Exon 8-1 Backward | TATATTTGGCCTCGTTGAAGTTGG |
| 19 | Exon 8-1 WT Forward **^3^** | CACAGGCTGTGAGCCCATTC **^5^** | 62 | 439 |
| 20 | Exon 8-1 WT Backward | CACCAGGGTTAGTTGCACAC |
| 21 | Exon 8-1 MUT Forward **^4^** | GGTTCAAGTATGTCACATTAGGC | 62 | 267 |
| 22 | Exon 8-1 MUT Backward | CCACTCAAGATAGCGCACAGA**^6^** |
| 23 | Exon 8-2 Forward | GAGCTGTACCTACTATGCCTTCAA | 59 | 622 |
| 24 | Exon 8-2 Backward | TGTTTCCTGCTCCTTTTCTTTCAA |
| 25 | Exon 8-3 Forward | CTCTTCCATTTCCCCCATGTCTTTC | 59 | 633 |
| 26 | Exon 8-3 Backward | GATTGGGGCTTATCACCTGGTTCC |
| 27 | Exon 9-1 Forward | AAGGCCTACAGCAGAGACAGCAGTT | 68 | 655 |
| 28 | Exon 9-1 Backward | AGGGACCGCAGCCCGTTCC |
| 29 | Exon 9-2 Forward | CCGCGATCCCGGATTTTCACG | 68 | 670 |
| 30 | Exon 9-2 Backward | TCCGAATCCTGGCCTGGAACATTTT |
| 31 | Exon 9-3 Forward (3’-UTR) | TGCTGCTGCCTTAAGTAGGAAGAG | 62 | 953 |
| 32 | Exon 9-3 Backward (3’-UTR) | ACGTAGGAGAAATAATGGCACCAA |
| 33 | Exon 9-4 Forward (3’-UTR) | TGTTACCTTCCACTTACTGTAGCA | 62 | 950 |
| 34 | Exon 9-4 Backward (3’-UTR) | GGGGGTATAATTCATTCCACAA |
| 35 | Exon 9-5 Forward (3’-UTR) | CAATCCCTTTTTACCACCAATAA | 62 | 1051 |
| 36 | Exon 9-5 Backward (3’-UTR) | TCTCCAGCTTTCACCCAGAAG |

**^1^***GRM1* gene accession # NM_000838.3 (NCBI)

**^2^**Forward and reverse primers designed for specific exons of *GRM1* gene

**^3^**WT: Allele specific primers designed for wild type allele

^4^MUT: Allele specific primers designed for mutant allele

**^5,6^**Sequence of primers with allele specificity at 3’end of primer
