# Supplementary material for: Identification of Novel GRM1 Mutations and Single Nucleotide Polymorphisms in Prostate Cancer Cell Lines and Tissues
Source: PLoS One. 2014 Jul 25;9(7):e103204. doi: 10.1371/journal.pone.0103204 (PMC4111546; doi:10.1371/journal.pone.0103204)
Supplement: Table S2 — Details of somatic mutations in GRM1 gene identified in whole exome or transcriptome sequencing studies of prostate cancer cell lines and tumors. (DOC) [file pone.0103204.s002.doc]

**Table S2:** Details of somatic mutations in *GRM1* gene identified in whole exome or transcriptome sequencing studies of prostate cancer cell lines and tumors.

| **S.N.** | **AA Change** | **Type** | **Predicted functional impact score^a^** | **3D^b^** | **Validation**  **Status^c^** | **COSMIC^d^** | **Prostate Cancer** | | **Study** |
| --- | --- | --- | --- | --- | --- | --- | --- | --- | --- |
|  |  |  |  |  |  |  | **Cell line** | **Tissue**  **Samples** |  |
| 1 | *G144S* | Missense | (-0.60) | 3D | V | 2 | 11 | 61 | [Grasso et al.](http://www.ncbi.nlm.nih.gov/pubmed/22722839) |
| 2 | *R297* | Nonsense | N/A |  | U | 21 | - | 300 | TCGA**^e^** |
| 3 | *A573E* | Missense | 0.5 |  | U | 1 | - | 112 | [Barbieri et al.](http://www.ncbi.nlm.nih.gov/pubmed/22610119) |
| 4 | *R681H* | Missense | 3.36 |  | U | 4 | - | 112 | [Barbieri et al.](http://www.ncbi.nlm.nih.gov/pubmed/22610119) |
| 5 | *R868H* | Missense | 2.21 |  | V | 1 | 11 | 61 | [Grasso et al.](http://www.ncbi.nlm.nih.gov/pubmed/22722839) |

**^a^**Based on evolutionary conserved pattern derived using combinatorial entropy formalism

**^b^**Crystal structure of protein is available

**^c^**V-Validated by alternative sequencing method or verified by independent PCR amplification and sequencing; U, unknown

**^d^**Overlapping mutations in COSMIC (Catalogue of somatic mutations in cancer)

**^e^**https://tcga-data.nci.nih.gov/tcga/
